# Antenatal Antibiotic Exposure Affects Enteral Feeding, Body Growth, and Neonatal Infection in Preterm Infants: A Retrospective Study

**DOI:** 10.3389/fped.2021.750058

**Published:** 2021-12-22

**Authors:** Ping Luo, Kun Zhang, You Chen, Xiuwen Geng, Tong Wu, Li Li, Ping Zhou, Ping-Ping Jiang, Liya Ma

**Affiliations:** ^1^School of Public Health, Sun Yat-sen University, Guangzhou, China; ^2^Department of Obstetrics, Bao'an Women and Children's Hospital, Jinan University, Shenzhen, China; ^3^Department of Neonatology, Bao'an Women and Children's Hospital, Jinan University, Shenzhen, China; ^4^Guangdong Provincial Key Laboratory of Food, Nutrition and Health, Sun Yat-sen University, Guangzhou, China; ^5^Department of Children Healthcare, Bao'an Women and Children's Hospital, Jinan University, Shenzhen, China

**Keywords:** antenatal antibiotic exposure, preterm infants, enteral feeding, body growth, neonatal infection

## Abstract

**Background:** Antibiotics are widely prescribed by obstetricians, which exposes a large number of infants to antenatal antibiotics (AAB). The effect of AAB on various aspects of neonatal development of preterm infants remains unclear.

**Methods:** In this retrospective study, infants born with gestational age (GA) between 22 ^+0^ and 36 ^+6^ weeks at our unit from 2017 to 2019 were included. Multivariable analysis was adopted to examine the associations between AAB exposure and various outcomes related to enteral feeding process, body growth, and neonatal infection after adjusting for potential confounders. Further subanalysis on the exposure level of AAB and stratified analysis by GA (<34 vs. ≥34 weeks) were also conducted.

**Results:** In this cohort comprising 2,543 preterm infants, AAB was associated with decreased risks of feeding intolerance (odds ratio [OR]: 0.63, 95% confidence interval [CI]: 0.48–0.82) and neonatal infection (OR: 0.63, 95% CI: 0.41–0.94). Higher AAB exposure level was associated with higher Z scores of birth weight (β = 0.37, 95% CI: 0.27–0.47), but lower Δbodyweight Z-scores (β = −0.20, 95% CI: −0.27 to −0.13). AAB was positively associated with the parameters related to body growth in infants with GA <34 weeks but negatively associated in those with GA ≥34 weeks.

**Conclusions:** AAB exposure affects the enteral feeding process and neonatal infection. The effects on body growth vary by the exposure level of AAB and GA of infants. A well-designed prospective and preferably multi-centre study with predefined parameters is required to confirm our findings.

## Introduction

Up to 40% of women receive antibiotics during pregnancy (1) mainly for treating infection of Group B streptococci (GBS), premature rupture of membranes (PROM), and prolonging pregnancy (2). This results in a large number of neonates having been exposed to antibiotics before birth. Reports have shown that while antenatal antibiotic (AAB) treatment reduces maternal morbidities, it is associated with increased risk of morbidities of infants immediately after birth or later in life, including very early onset inflammatory bowel disease (IBD) (3), infant asthma (4), and childhood obesity (5).

Thus far, most studies on AAB affecting offspring development have focused on the general neonatal population, with only few studies being conducted in preterm infants, where AAB likely exacerbated the risk of prematurity on development. A report has shown that AAB exposure is associated with higher risk of gut-related morbidities in preterm infants, including necrotising enterocolitis (NEC) (6, 7). AAB also affects the colonization of the fetal and neonatal gut, thereby compromising the gut microbiome in preterm infants (8). This compromised gut microbiome is involved in impaired gut development (9) and NEC, affecting the progress of enteral feeding (10) and, consequently, body growth. Studies have shown that rapid enteral feeding progress, including early initiation (11), fast advancement of feeding rate (12, 13), and early attainment of full enteral feeding (14), are associated with early regaining of birth weight and decreased incidence of extrauterine growth restriction in preterm infants. An association was also found between rapid growth in the first postnatal weeks and neurocognitive benefits in later life in very preterm infants (15), indicating the effect of fast enteral feeding on long-term development. Neonatal infection and sepsis have been linked to mortality and adverse outcomes of different organs of infants (16); however, how AAB affects neonatal infection is yet uncertain (17, 18). In general, how AAB exposure affects enteral feeding, body growth, and infection in preterm infants remains inconclusive. Because the rates of cesarean section and GBS colonization requiring AAB treatment are continuing to rise (2), there is an urgent need to define the effects of AAB exposure on preterm infants.

Herein, we evaluated the relationship between AAB exposure and enteral feeding, body growth, and infection in preterm infants born between 2017 and 2019 at our hospital.

## Methods

### Study Population

This retrospective cohort study was conducted at the Shenzhen Bao'an Women and Children's Hospital, Shenzhen, China. The research ethics committees of the School of Public Health, Sun Yat-sen University, and the study hospital approved the study (permit no. 2019-148 and LISCHY2019-10-04-01, respectively).

Preterm infants born with gestational age (GA) between 22 ^+0^ and 36 ^+6^ weeks from January 1, 2017, to December 31, 2019, at the study hospital were included. The exclusion criteria were death before hospital discharge, major congenital anomalies, admission to the neonatal department later than 24 h after birth, and incomplete neonatal information or maternal information.

### AAB Exposure and Outcomes

Indications for antibiotic treatment in pregnant women included cesarean section, preterm premature rupture of membranes (PPROM), and GBS infection of the lower genital tract. Local practice of prepartum AAB treatment is attached as supplementary information (Supplementary Table S1). AAB exposure was defined as receipt of minimum one dose of any type of antibiotic from admission to the obstetric unit until delivery. Infants of mothers receiving AAB treatment were assigned to the AAB group, while other infants were assigned to the No AAB group. Relevant maternal and neonatal information of eligible infants was extracted from the electronic medical and nursing record system.

The primary outcome was the incidence of feeding intolerance, defined as ≥3 episodes of “excessive” gastric residuals with volume exceeding 30% of the previous meal within one calendar day (19). Secondary outcomes were related to enteral feeding progress, body growth, and neonatal infection. Enteral feeding progress included the following parameters: (1) TIEF, the time from birth to the initiation of enteral feeding (the time interval from birth to the first calendar day with eight consecutive enteral feedings in days); (2) TFEF120, the time from the initiation of enteral feeding to full enteral feeding of 120 ml/kg bodyweight/day; (3) the incidence of attaining full enteral feeding before hospital discharge; and (4) the advancement rate of enteral feeding (ml/kg/day, the difference in the volumes between the initiation and attainment of full enteral feeding divided by the number of calendar days). The outcomes related to body growth included (1) the incidence of regaining birth weight before discharge; (2) the time from birth to regaining birth weight (TRBW, in calendar days); (3) the weight velocity [g/kg/day, calculated by the exponential method (20) from birth to postnatal age of 14 days or discharge, whichever came first]; and (4) the Z scores of bodyweights at birth and on discharge, and Δbodyweight Z-score. All Z scores were calculated based on the Fenton's growth chart for preterm infants (21). Clinical infection was defined as ≥2 of the following: elevated or decreased counts of white blood cells (≥30 × 10^9^/l in the first 3 days of life, ≥20 × 10^9^/l after 3 days since birth, or <5 × 10^9^/l on any day) (22, 23); decreased platelet count (<100 × 10^9^/l) (24); elevated level of C-reactive protein (CRP, ≥10 mg/l) (25); and elevated level of procalcitonin (PCT, ≥0.5 mg/l) (26). The time to first infection was defined as the time from birth to the start of the first episode of clinical infection based on the above criteria, in calendar days. The number of infection episodes was defined as the total number of infection episodes during the hospitalization.

For confounder adjustment, maternal infection was defined as chorioamnionitis and/or PPROM during pregnancy. A composite parameter, adverse neonatal condition, was defined as at least one of the following: gastrointestinal tract (GIT)-related diseases (gastrointestinal perforation, gastrointestinal bleeding, food-induced enterocolitis, or NEC); respiratory distress syndrome (RDS); bronchopulmonary dysplasia (BPD); retinopathy of prematurity (ROP); sepsis; and brain injury (intraventricular hemorrhage or periventricular leukomalacia). Detailed diagnostic criteria of these conditions and diseases are listed in Supplementary Table S2. Proportion of own mother's milk (OMM) in enteral feeding was calculated by dividing the volume of OMM by that of all enteral feeds throughout the observation period and divided into three levels, namely, no OMM, mixed feeding, and exclusive OMM.

### Statistical Analysis

Maternal and neonatal characteristics were compared between the AAB and No AAB groups using Student's *t*-test for normally distributed continuous variables, Wilcoxon rank-sum test for continuous non-parametric variables, and Chi-square test for categorical variables.

Multivariable analysis was used to assess the association of AAB and various outcomes with adjustment for different confounders using the No AAB group as reference. The bodyweight Z-score at birth and on discharge, Δbodyweight Z-score, advancement rate of enteral feeding, and weight velocity were tested with multivariate linear regression. A β-regression coefficient, 95% confidence intervals (CIs), and a *p*-value from the F test were reported for each outcome. Time-to-event data including TIEF, TFEF120, TRBW, and the time to the first infection were tested with the Cox proportional hazard model. A hazard ratio (HR) was reported together with 95% CIs and a *p*-value calculated using the Wald test. Incidences of feeding intolerance, attainment of full enteral feeding, regaining birth weight before discharge, sepsis, and clinical infection were tested with logistic regression. Odds ratio (OR), 95% CIs, and *p*-value using the chi-square test were calculated. Clinical infection episode was tested as recurrent time-to-event data with the Prentice, Williams, and Peterson total time (PWP-TT) model (27). The length of hospital stay was not included in the PWP-TT model as it was highly collinear with GA.

Further subanalysis was conducted in the AAB group according to the days of treatment (DOT), ≤ 3 vs. >3 days, with the ≤ 3-day subgroup as reference. DOT was calculated as the sum of the days of antenatal exposure for each antibiotic used (28). A stratified analysis was conducted on the whole population stratified by GA, <34 vs. ≥34 weeks. Relevant β-coefficients, HRs, and ORs as well as *p*-values for both analyses were calculated as described above.

For confounder adjustment, only GA, sex, and delivery mode were adjusted in Model I. Maternal factors, adverse neonatal condition, and proportion of OMM were further adjusted in Model II based on Model I. Refer to each table for the factors included in different models.

All statistical tests were two-sided with a significant threshold of 0.05. All data management and statistical analyses were conducted in R (29) interfaced with R studio (30) using the R package *survival* (31).

## Results

**Figure 1:**
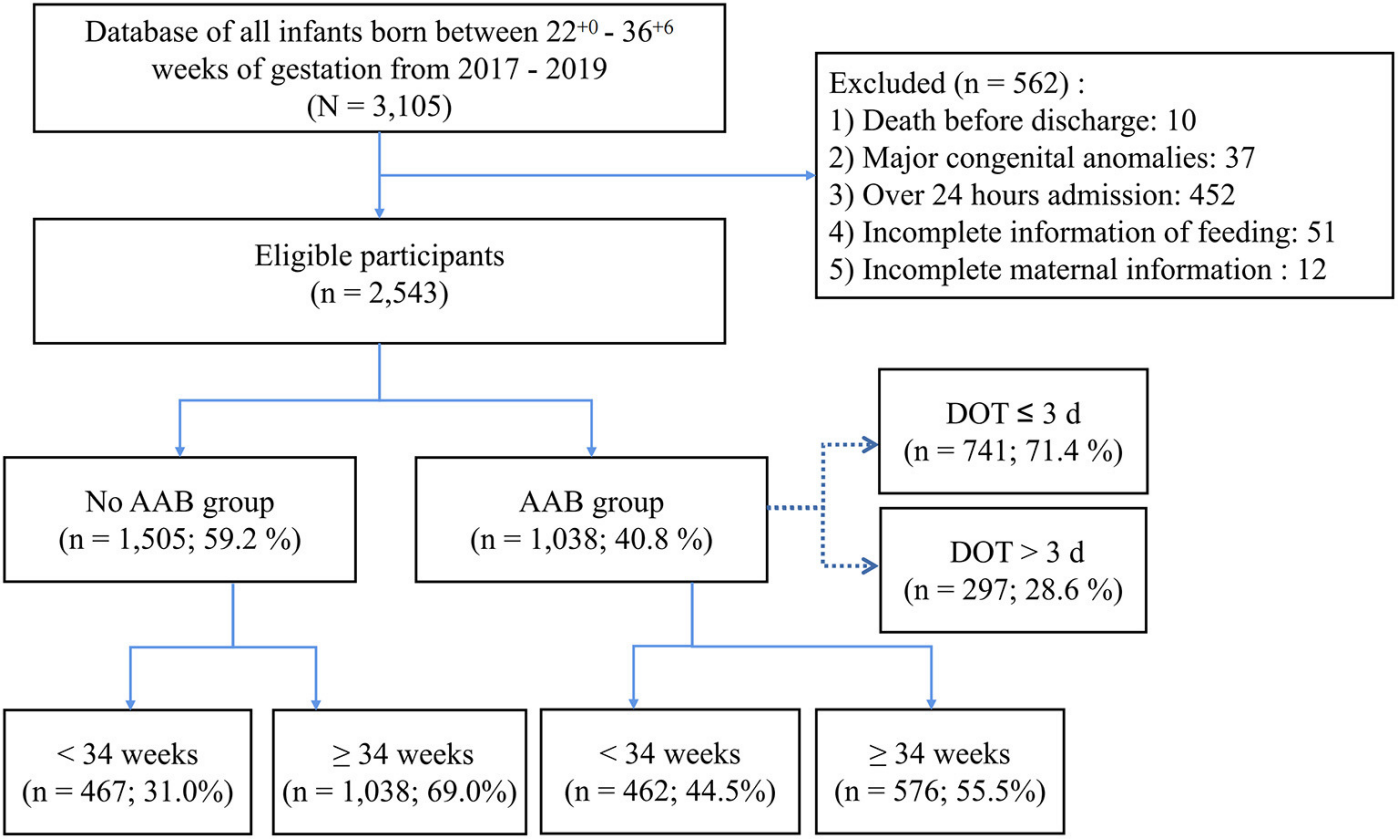
Preterm Infants included in the study.

A total of 3,105 preterm infants were identified during the study period, and 2,543 of them were included in the study (Figure 1). Characteristics of the included infants and their mothers are shown in Table 1 and Supplementary Table S3, respectively. Compared with the No AAB infants, the AAB ones were born earlier (median GA: 34.3 vs. 35.0 weeks, *p* < 0.001) and with higher bodyweight Z-scores (mean: −0.3 vs. −0.4, *p* < 0.01). The AAB infants had a trend toward higher incidence of feeding intolerance than the No AAB ones (25.1 vs. 21.9%, *p* = 0.06), significantly slower advancement of enteral feeding (mean: 15.6 vs. 16.6 ml/kg/d, *p* < 0.001), and higher incidence of attaining full enteral feeding before discharge (70.1 vs. 66.2%, *p* = 0.04). Significantly higher bodyweight Z-scores were found in the AAB infants than in the No AAB ones, at birth (mean: −0.3 vs. −0.4, *p* < 0.01), while no significant difference was found in the bodyweight Z-scores on discharge or in Δbodyweight Z-scores. No significant difference was found in the incidence of neonatal infection between the AAB and No AAB infants, but the AAB infants had longer time to first infection (median: 4.0 vs. 3.0 days, *p* = 0.04).

**Table 1:** Baseline characteristics and neonatal outcomes of the no AAB and AAB groups.

**Outcomes**	**No AAB group (*n =* 1,505)**	**AAB group (*n =* 1,038)**	**p**
**At birth**
Male sex, yes, *n* (%)	816 (54.2)	606 (58.4)	**0.04^a^**
GA, weeks, median (IQR)	35.0 (33.4–35.9)	34.3 (32.0–36.0)	**<0.001^b^**
Small for gestational age, yes, *n* (%)	234 (15.5)	96 (9.2)	**<0.001^a^**
Large for gestational age, yes, *n* (%)	25 (1.7)	17 (1.6)	1.00^a^
Bodyweight, g, mean ± SD	2128.7 ± 494.1	2000.8 ± 551.0	**<0.001^c^**
Bodyweight Z-score, mean ± SD	−0.4 ± 0.8	−0.3 ± 0.8	**<0.01^c^**
Body length^d^, cm, median (IQR), (*n =* 1,498 vs. *n =* 1,030)	46.0 (43.0–48.0)	45.0 (42.0–47.0)	**<0.001^b^**
Body length Z-score, mean ± SD	0.1 ± 1.0	0.1 ± 1.0	0.29^c^
Head circumference^d^, cm, median (IQR), (*n =* 1,496 vs. *n =* 1,029)	32.0 (30.0–33.0)	31.0 (29.0–33.0)	**<0.001^b^**
Head circumference Z-score, mean ± SD	0.1 ± 1.0	0.1 ± 1.0	0.84^c^
**During hospitalization**
TIEF, days, median (IQR)	1.0 (1.0–3.0)	1.0 (1.0–3.0)	0.08^b^
Advancement rate of enteral feeding^e^, mean ± SD, ml/kg/day, (*n =* 1,490 vs. *n =* 1,030)	16.6 ± 6.5	15.6 ± 6.4	**<0.001^c^**
Feeding intolerance, yes, *n* (%)	329 (21.9)	261 (25.1)	0.06^a^
Attaining full enteral feeding before discharge, yes, *n* (%)	997 (66.2)	728 (70.1)	**0.04^a^**
TFEF120, days, median (IQR)	6.0 (5.0–8.0)	6.0 (5.0–9.0)	**0.001^b^**
Regaining birth weight before discharge, yes, *n* (%)	940 (62.5)	678 (65.3)	0.15^a^
TRBW, days, median (IQR)	7.0 (4.0–10.0)	7.0 (4.0–10.0)	0.59^b^
Weight velocity, g/kg/day, mean ± SD	−0.4 ± 6.6	0.7 ± 7.1	**<0.001^c^**
Proportion of OMM, yes, *n* (%)			**<0.05^a^**
None	566 (37.6)	344 (33.1)	
Mixed feeding	668 (44.4)	480 (46.2)	
Exclusive	271 (18.0)	214 (20.6)	
Adverse neonatal condition, yes, *n* (%)	225 (15.0)	199 (19.2)	**<0.01^a^**
GIT-related diseases, yes, *n* (%)	70 (4.7)	47 (4.5)	0.96^a^
NEC, yes, *n* (%)	16 (1.1)	11 (1.1)	0.17^a^
RDS, yes, *n* (%)	66 (4.4)	41 (3.9)	0.66^a^
BPD, yes, *n* (%)	40 (2.7)	83 (8.0)	**<0.001^a^**
ROP, yes, *n* (%)	17 (1.1)	47 (4.5)	**<0.001** ^a^
Sepsis, yes, *n* (%)	98 (6.5)	90 (8.7)	**<0.05^a^**
Clinical infection, yes, *n* (%)	95 (6.3)	72 (6.9)	0.59^a^
Time to 1st infection, days, median (IQR), (*n =* 95 vs. *n =* 72)	3.0 (1.0–6.0)	4.0 (2.0–12.3)	**0.04^b^**
Clinical infection episodes, times median (IQR), (*n =* 95 vs. *n =* 72)	1.0 (1.0–1.5)	1.0 (1.0–2.0)	**<0.05^b^**
**Upon discharge**
Postmenstrual age, weeks, median (IQR)	36.9 (36.0–37.6)	36.7 (35.9–37.4)	0.05^b^
Bodyweight, g, mean ± SD	2300.6 ± 338.5	2301.7 ± 338.6	0.94^c^
Bodyweight Z-score, mean ± SD	−1.3 ± 0.9	−1.3 ± 0.8	0.08^c^
Δbodyweight Z-score, mean ± SD	−0.9 ± 0.5	−0.9 ± 0.6	0.13^c^
Length of hospital stay, days, median (IQR)	10.0 (7.0–22.0)	11.0 (7.0–30.8)	**<0.001^b^**

a *Tested with the Chi-square test*.

b *Tested with the Wilcoxon rank-sum test*.

c *Tested with Student's t test*.

d *Data missing with respect to body length and head circumference*.

e *Twenty-three subjects discharged when TIEF was initiated, and the rate of enteral feeding advancement could not be calculated. Bold indicates significant differences (p < 0.05). AAB, antenatal antibiotics; BPD, bronchopulmonary dysplasia; GA, gestational age; GIT, gastrointestinal tract; IQR, interquartile range; NEC, necrotizing enterocolitis; OMM, own mother's milk; RDS, respiratory distress syndrome; ROP, retinopathy of prematurity; SD, standard deviation; TFEF120, time to full enteral feeding of 120 ml/kg/day; TIEF, time to the initiation of enteral feeding; TRBW, time to regain birth weight*.

Results of the multivariable analyses are listed in Tables 2–4. AAB exposure was significantly associated with a lower risk of feeding intolerance in the whole study population (OR: 0.63, 95% CI: 0.48–0.82, *p* < 0.001) and in the subgroup with GA ≥34 weeks (OR: 0.55, 95% CI: 0.33–0.89, *p* = 0.02), but not in the subgroup with GA <34 weeks. No association between AAB DOT and the incidence of feeding intolerance was found (Table 3). Enteral feeding process-wise, AAB exposure was associated with shorter TIEF in the whole study population (HR: 1.16, 95% CI: 1.07–1.26, *p* < 0.001) and in the subgroup of GA ≥34 weeks (HR: 1.19, 95% CI: 1.07–1.32, *p* < 0.01). No significant association was found for the advancement rate of enteral feeding, the incidence of attaining full enteral feeding, or TFEF120 in the whole study population or in either GA subgroup. No significant association was found for AAB DOT with these parameters in the whole population or in the GA subgroups.

**Table 2:** Associations of AAB exposure and neonatal outcomes related to enteral feeding process, body growth, and infection.

**Outcomes**	**Model I^a^**		**Model II^b^**	
	**β/OR/HR (95% CI)**	**p**	**β/OR/HR (95% CI)**	**p**
Feeding intolerance, OR	0.62 (0.49–0.80)	**<0.001**	0.63 (0.48–0.82)	**<0.001**
**Enteral feeding**
TIEF, HR	1.19 (1.10–1.29)	**<0.001**	1.16 (1.07–1.26)	**<0.001^c^**
Advancement rate of enteral feeding ^d^, (n = 1,490 vs. n = 1,030), β	0.11 (−0.34–0.55)	0.63	0.02 (−0.44–0.47)	0.94
Attaining full enteral feeding before discharge, OR	0.97 (0.80–1.17)	0.74	0.99 (0.81–1.21)	0.92
TFEF120, HR	1.07 (0.97–1.18)	0.18	1.02 (0.92–1.13)	0.68
**Body growth**
Regaining birth weight before discharge, OR	0.88 (0.73–1.06)	0.18	0.95 (0.78–1.15)	0.57
TRBW, HR	1.06 (0.95–1.17)	0.30	1.08 (0.97–1.20)	0.15
Weight velocity, β	0.20 (−0.30–0.70)	0.44	0.26 (−0.26–0.78)	0.32
Bodyweight Z-score at birth ^e^, β	0.09 (0.03–0.16)	**<0.01**	0.03 (−0.03–0.10)	0.33^f^
Bodyweight Z-score on discharge ^e^, β	0.06 (−0.01–0.13)	0.08	0.05 (−0.02–0.12)	0.17
Δbodyweight Z-score ^e^, β	−0.03 (−0.07–0.01)	0.13	0.02 (−0.02–0.06)	0.27
**Infection**
Sepsis, OR	0.71 (0.50–1.00)	0.06	0.84 (0.57–1.22)	0.37^g^
Clinical infection, OR	0.52 (0.35–0.75)	**<0.001**	0.63 (0.41–0.94)	**0.03^g^**
Time to 1st infection, HR	0.53 (0.38–0.74)	**<0.001**	0.63 (0.44–0.91)	**0.01^g^**
Clinical infection episodes, HR	0.68 (0.51–0.91)	**<0.01**	0.81 (0.58–1.14)	0.23^g^

a *Adjusted for GA, sex, and delivery mode unless otherwise specified*.

b *Adjusted for GA, sex, delivery mode, maternal age, ACS, maternal diabetes, maternal hypertension, adverse neonatal condition, and proportion of OMM unless otherwise specified*.

c *Adjusted for GA, sex, delivery mode, maternal age, ACS, maternal diabetes, and maternal hypertension unless otherwise specified*.

d *Twenty-three subjects discharged when TIEF was initiated and the rate of enteral feeding advancement could not be calculated*.

e *GA and sex were not adjusted in any of these outcomes*.

f *Adjusted for delivery mode, maternal age, ACS, maternal diabetes, and maternal hypertension unless otherwise specified*.

g *Adjusted for GA, sex, delivery mode, maternal age, ACS, maternal diabetes, maternal hypertension, proportion of OMM, and maternal infection. Bold indicates significant differences (p < 0.05). AAB, antenatal antibiotics; ACS, antenatal corticosteroids; CI, confidence interval; GA, gestational age; HR, hazard ratio; OMM, own mother's milk; OR, odds ratio; TFEF120, time to full enteral feeding of 120 ml/kg/day; TIEF, time to the initiation of enteral feeding; TRBW, time to regain birth weight*.

**Table 3:** Associations of DOT of AAB exposure and neonatal outcomes related to enteral feeding process, body growth, and infection.

**Outcomes**	**Model I^a^**		**Model II^b^**	
	**β/OR/HR (95% CI)**	**p**	**β/OR/HR (95% CI)**	**p**
Feeding intolerance, OR	1.12 (0.76–1.66)	0.56	1.13 (0.75–1.70)	0.56
**Enteral feeding**
TIEF, HR	1.01 (0.87–1.17)	0.94	0.97 (0.84–1.13)	0.73^c^
Advancement rate of enteral feeding^d^, (*n =* 735 vs. *n =* 295), β	0.39 (-0.40–1.19)	0.33	0.41 (-0.38–1.21)	0.31
Attaining full enteral feeding before discharge, OR	1.03 (0.68–1.58)	0.88	1.10 (0.72–1.69)	0.66
TFEF120, HR	1.13 (0.95–1.34)	0.17	1.15 (0.97–1.38)	0.11
**Body growth**
Regaining birth weight before discharge, OR	0.85 (0.55–1.32)	0.47	0.85 (0.54–1.32)	0.46
TRBW, HR	1.04 (0.87–1.23)	0.70	1.01 (0.85–1.21)	0.90
Weight velocity, β	−0.06 (-1.03–0.91)	0.91	−0.14 (-1.12–0.84)	0.78
Bodyweight Z-score at birth^e^, β	0.41 (0.31–0.51)	**<0.001**	0.37 (0.27–0.47)	**<0.001^f^**
Bodyweight Z-score on discharge^e^, β	0.08 (-0.03–0.18)	0.18	0.13 (0.02–0.24)	**0.02**
Δbodyweight Z-score^e^, β	−0.33 (-0.40 to−0.26)	**<0.001**	−0.20 (-0.27 to−0.13)	**<0.001**
**Infection**
Sepsis, OR	1.21 (0.72–2.01)	0.47	1.35 (0.78–2.36)	0.29^g^
Clinical infection, OR	0.96 (0.54–1.69)	0.89	1.15 (0.62–2.12)	0.66^g^
Time to 1st infection, HR	0.93 (0.56–1.53)	0.77	1.08 (0.63–1.83)	0.78^g^
Clinical infection episodes, HR	0.89 (0.59–1.35)	0.59	0.97 (0.62–1.51)	0.90^g^

a *Adjusted for GA, sex, and delivery mode unless otherwise specified*.

b *Adjusted for GA, sex, delivery mode, maternal age, ACS, maternal diabetes, maternal hypertension, adverse neonatal condition, and proportion of OMM unless otherwise specified*.

c *Adjusted for GA, sex, delivery mode, maternal age, ACS, maternal diabetes, and maternal hypertension unless otherwise specified*.

d *Eight subjects discharged when TIEF was initiated and the rate of enteral feeding advancement could not be calculated*.

e *GA and sex were not adjusted in any of these outcomes*.

f *Adjusted for delivery mode, maternal age, ACS, maternal diabetes, and maternal hypertension unless otherwise specified*.

g *Adjusted for GA, sex, delivery mode, maternal age, ACS, maternal diabetes, maternal hypertension, proportion of OMM, and maternal infection. Bold indicates significant differences (p < 0.05). AAB, antenatal antibiotics; ACS, antenatal corticosteroids; CI, confidence interval; DOT, days of treatment; GA, gestational age; HR, hazard ratio; OMM, own mother's milk; OR, odds ratio; TFEF120, time to full enteral feeding of 120 mL/kg/day; TIEF, time to the initiation of enteral feeding; TRBW, time to regain birth weight*.

**Table 4:** Associations of AAB exposure and neonatal outcomes related to enteral feeding process, body growth, and infection-stratified analysis by GA.

**Outcomes**	** <34 weeks (***n** **=*** 929)**				**≥34 weeks (***n** **=*** 1,614)**			
	**Model I^a^**		**Model II^b^**		**Model I^a^**		**Model II^b^**	
	**β/OR/HR (95% CI)**	**p**	**β/OR/HR (95% CI)**	**p**	**β/OR/HR (95% CI)**	**p**	**β/OR/HR (95% CI)**	**p**
Feeding intolerance, OR	0.91 (0.70–1.19)	0.49	0.90 (0.66–1.22)	0.50	0.60 (0.37–0.93)	**0.03**	0.55 (0.33–0.89)	**0.02**
**Enteral feeding**
TIEF, HR	1.02 (0.89–1.16)	0.79	0.91 (0.79–1.05)	0.21^c^	1.17 (1.06–1.30)	**<0.01**	1.19 (1.07–1.32)	**<0.01^c^**
Advancement rate of enteral feeding^d^, β	−0.41 (−1.19–0.37)	0.30	−0.62 (−1.39–0.14)	0.11	−0.07 (−0.65–0.51)	0.81	−0.04 (−0.63–0.55)	0.89
Attainment of full enteral feeding before discharge, OR	0.78 (0.46–1.31)	0.35	0.89 (0.50–1.55)	0.67	0.95 (0.77–1.16)	0.60	0.94 (0.76–1.17)	0.58
TFEF120, HR	0.89 (0.78–1.02)	0.08	0.90 (0.78–1.05)	0.17	1.06 (0.92–1.22)	0.40	1.02 (0.88–1.19)	0.75
**Body growth**
Regaining birth weight before discharge, OR	2.34 (1.47–3.79)	**<0.001**	2.58 (1.56–4.34)	**<0.001**	0.71 (0.57–0.87)	**<0.001**	0.72 (0.58–0.90)	**<0.01**
TRBW, HR	1.38 (1.21–1.59)	**<0.001**	1.41 (1.22–1.63)	**<0.001**	0.81 (0.70–0.95)	**<0.01**	0.83 (0.71–0.97)	**0.02**
Weight velocity, β	1.40 (0.61–2.19)	**<0.001**	1.62 (0.80–2.45)	**<0.001**	−0.19 (−0.85–0.46)	0.57	−0.23 (−0.90–0.44)	0.50
Bodyweight Z–score at birth^e^, β	0.14 (0.04–0.23)	**0.01**	0.05 (−0.04–0.15)	0.29^f^	−0.02 (−0.10–0.06)	0.64	−0.04 (−0.13–0.04)	0.32^f^
Bodyweight Z-score on discharge^e^, β	0.21 (0.10–0.32)	**<0.001**	0.13 (0.01–0.24)	**0.03**	0.01 (−0.08–0.10)	0.78	−0.01 (−0.10–0.08)	0.80
Δbodyweight Z–score^e^, β	0.07 (−0.01–0.15)	0.06	0.07 (−0.01–0.15)	0.07	0.03 (−0.01–0.07)	0.07	0.03 (−0.01–0.07)	0.08
**Infection**
Sepsis, OR	1.17 (0.83–1.67)	0.37	1.42 (0.94–2.15)	0.10^g^	0.52 (0.22–1.11)	0.11	0.57 (0.24–1.25)	0.18^g^
Clinical infection, OR	0.91 (0.63–1.33)	0.64	1.11 (0.72–1.72)	0.65^g^	0.51 (0.21–1.07)	0.09	0.54 (0.23–1.17)	0.14^g^
Time to 1st infection, HR	0.86 (0.61–1.22)	0.41	1.01 (0.67–1.51)	0.97^g^	0.54 (0.25–1.20)	0.13	0.60 (0.27–1.35)	0.22^g^
Clinical infection episodes, HR	0.94 (0.70–1.27)	0.68	1.12 (0.78–1.61)	0.53^g^	0.64 (0.30–1.38)	0.25	0.71 (0.33–1.51)	0.38^g^

a *Adjusted for GA, sex, and delivery mode unless otherwise specified*.

b *Adjusted for GA, sex, delivery mode, maternal age, ACS, maternal diabetes, maternal hypertension, adverse neonatal condition, and proportion of OMM unless otherwise specified*.

c *Adjusted for GA, sex, delivery mode, maternal age, ACS, maternal diabetes, and maternal hypertension unless otherwise specified*.

d *Eight subjects discharged when TIEF was initiated and the rate of enteral feeding advancement could not be calculated*.

e *GA and sex were not adjusted in any of these outcomes*.

f *Adjusted for delivery mode, maternal age, ACS, maternal diabetes, and maternal hypertension unless otherwise specified*.

g *Adjusted for GA, sex, delivery mode, maternal age, ACS, maternal diabetes, maternal hypertension, proportion of OMM, and maternal infection. Bold indicates significant differences (p < 0.05). AAB, antenatal antibiotics; ACS, antenatal corticosteroids; CI, confidence interval; GA, gestational age; HR, hazard ratio; OMM, own mother's milk; OR, odds ratio; TFEF120, time to full enteral feeding of 120 ml/kg/day; TIEF, time to the initiation of enteral feeding; TRBW, time to regain birth weight*.

At birth, AAB exposure was associated with larger bodyweight Z-score (β = 0.09, 95% CI: 0.03–0.16, *p* < 0.01) if only delivery mode, sex, and GA were adjusted (Table 2). No significant association with AAB exposure was observed for the bodyweight Z-score on discharge, Δbodyweight Z-score, or weight velocity in the whole study population. With the AAB DOT ≤ 3 days as reference, DOT of AAB >3 days was associated with larger bodyweight Z-score at birth (β = 0.37, 95% CI: 0.27–0.47, *p* < 0.001) and on discharge (β = 0.13, 95% CI: 0.02–0.24, *p* = 0.02), and lower Δbodyweight Z-score (β = −0.20, 95% CI: −0.27 to −0.13, *p* < 0.001) (Table 3). In the subgroups with different GAs, opposite associations were found for the incidence of regaining birth weight, TRBW, and weight velocity (Table 4). AAB exposure was associated with increased incidence of regaining birth weight and shorter TRBW in the subgroup of GA <34 weeks but negatively associated with these two outcomes in the subgroup of GA ≥34 weeks. Only in the subgroup of GA <34 weeks was AAB exposure associated with larger weight velocity (β = 1.62, 95% CI: 0.80–2.45, *p* < 0.001). Only in the subgroup of GA ≥34 weeks was AAB associated with increased Δbodyweight Z-score (β = 0.04, 95% CI: 0.01–0.07, *p* = 0.04) before the adverse neonatal condition and the proportion of OMM were included in the model.

AAB exposure was associated with lower risk of neonatal infection (OR: 0.63, 95% CI: 0.41–0.94, *p* = 0.03) and longer time to the first infection (HR: 0.63, 95% CI: 0.44–0.91, *p* = 0.01), but not with the incidence of sepsis or the number of neonatal infection episodes in the whole study population (Table 2). No significant association was found in neonatal infection-related parameters in any subgroups with different GA or AAB DOT.

## Discussion

In this retrospective study, whether and how AAB exposure affects enteral feeding progress, body growth, and neonatal infection were investigated in preterm infants based on a cohort at our department.

Feeding intolerance is a negative functional marker of the neonatal gut (32). Reduced feeding intolerance associated with AAB exposure after adjustment for confounders suggests that AAB may independently improve the functional development of the gut in preterm infants. This could be attributed to the fact that AAB dampened the maternal infection-related inflammation, thus limiting the adverse effect on the fetal gut. However, a report on neonatal pigs suggested otherwise. AAB for 1 week before farrowing delayed gut development in terms of structure and enzymatic activities in newborn pigs (33). AAB also reduces the diversity of the neonatal gut microbiome (34), which in turn can affect the neonatal gut development and feeding intolerance (10). How AAB affects gut development before and after birth mechanistically requires further investigations. Absence of association with feeding intolerance in infants at smaller GA highlights the uniqueness of this subpopulation; no detrimental effect of AAB was found. In many cases, NEC is developed post severe, malign feeding intolerance and is the most serious morbidity of the neonatal gut. A multicentre retrospective study showed that AAB exposure reduced the risk of NEC (35), while the ORACLE trial showed an increased rate of NEC post treatment with amoxicillin–clavulanic acid (6); thus, it is still undetermined how AAB affects NEC. Because of a limited number of NEC cases (27), there is not enough statistical power to assess whether AAB affects the NEC incidence in our study. A study with a relatively larger number of NEC cases is needed to assess this relationship.

In clinical practice, feeding intolerance often disturbs the enteral feeding progress (36). However, the enteral feeding process was not affected by AAB in the whole study population and in both GA subgroups, suggesting that the feeding intolerance cases found in our study was probably benign in nature. The only association with earlier initiation of enteral feeding suggests that the effect of AAB is more prominent on fetal gut development than on the neonatal gut development. This is similar to the findings in preterm pigs with antenatal exposure to lipopolysaccharide (LPS), a bacterial endotoxin, where the antenatal exposure mainly affected the fetal gut development (37). Our subanalyses on AAB DOT further demonstrated that the exposure level of AAB did not affect the feeding-related parameters.

The aim of assuring fast increment of enteral feeding is to provide sufficient nutrients and energy for preterm infants to meet their increasing nutritional needs. Few studies have examined how AAB affects neonatal body growth. In this study, AAB did not affect the parameters related to body growth adopted in the whole study population, which is unlike the neonatal antibiotic exposure. The disappearance of significance on the bodyweight Z-score at birth after the adjustment for maternal factors suggests that AAB affecting birth weight was dependent on other maternal factors. Further, the exposure level of AAB, shown as AAB DOT, affected body growth. High exposure level of AAB (DOT >3 days) conferred benefits to the body growth of fetuses but limited their neonatal body growth; this effect was independent of other maternal and neonatal factors. Preterm infants with different GAs responded to AAB differently in terms of body growth. AAB was associated with lower incidence and longer time of regaining birth weight in infants of GA ≥34 weeks, while higher incidence and shorter time were seen in infants of GA <34 weeks, suggesting that AAB could confer benefits to the infants with small GA possibly *via* diminishing the effect of maternal infection. However, both subgroups reached a trend toward positive association with Δbodyweight Z-scores, despite low coefficients, suggesting marginal but beneficial effect of AAB on body growth during hospitalization. A further test also showed that adverse neonatal condition was the reason for the disappearance of significance regarding the association of Δbodyweight Z-scores in the subgroup of GA ≥34 weeks (data not shown), suggesting that the effect of AAB is dependent on conditions included in this composite parameter.

Preterm infants are predisposed to infection, and it is affected by many factors including maternal infection as shown in a meta-analysis (38). In this study, maternal infection was accounted for among other adverse maternal conditions. Even after this adjustment, AAB was associated with a lower risk of clinical infection, suggesting that AAB reducing the risk of neonatal infection is independent of maternal infection. Our results are in accordance with those from a meta-analysis where AAB was associated with reduced neonatal clinical infection in preterm labor (17). Mechanistically, this could be attributed to the effect of AAB on the fetal or neonatal microbiome, given its important roles in immunity development of preterm infants. Similar to our finding on doctor-diagnosed sepsis, two meta-analyses reported antibiotic prophylaxis during the second and third trimesters of pregnancy (39), and AAB for preterm labor with intact membrane were not associated with reduced incidence of neonatal sepsis (17). AAB was associated with longer time to the first infection, but not with the infection episodes, which implies that AAB mainly affects the fetal immunity development; thus, its effect is limited to the immediate neonatal period. In our study, the study population was divided based on GA 34 weeks for subanalysis of different GA groups, partly due to the limited number of early preterm infants (GA <28 weeks, *n* = 86). Early preterm infants are assumed to be more seriously affected by AAB, but few studies have focused on this group, possibly because of the limited number of cases and the complicated condition of these infants and the treatments received by them and their mothers. A much larger cohort would render an opportunity for a more focused assessment of the infants of smaller GA (<28 weeks). The DOT of AAB did not affect neonatal infection in the AAB infants, suggesting that the overall exposure level of AAB might only have limited effect. However, how different types of antibiotics affect neonatal infection remains unclear, despite the ORACLE trial suggesting erythromycin over amoxicillin–clavulanic acid therapy. Although our results suggest that AAB only affects neonatal infection to a limited extent, a population-based cohort study showed that AAB exposure could increase the risk of infection in childhood (40). This requires further investigations on the infection susceptibility of preterm infants with AAB exposure in short and long terms.

The merits of this study include the very specific population being analyzed—preterm infants—which are relatively homogenous. Based on the electronic medical and nursing records, nearly complete information of antibiotic usage and details of enteral feeding was available to ensure relatively accurate and reliable readouts of the analyzed related parameters. The main limitation of the current study is the potential bias due to the single-center and retrospective nature. Body length and head circumference are two other parameters for assessing body growth of infants, even preferred by some researchers (41, 42), but complete data of the study population on discharge were not available in some cases, which precluded further analysis. Estimating feeding intolerance only based on the feeding charts is extremely difficult. Thus, the definition of feeding intolerance adopted here was based on the volumes of gastric residuals; no quality data such as color or consistency were included as they are not routinely recorded at out unit. These features have been suggested to be of use in differentiating benign and adverse gastric residuals (43). To adjust for known confounding factors from the maternal and neonatal sides, two composite parameters—maternal infection and adverse neonatal condition—were adopted. However, residual confounding from other factors cannot be ruled out. In this study, antibiotics on the day of delivery were not included as the antibiotics used before and after delivery could not be differentiated, but reports showed that intrapartum antibiotics affect gut dysbiosis and increase the risk of early onset sepsis in term infants (44). Besides, the types of antibiotics used were not assessed, but studies have shown that different types of antibiotics may pose different effect on the fetuses (18, 45).

In conclusion, our study provided preliminary evidence for AAB exposure affecting enteral feeding, body growth, and neonatal infection in preterm infants. The exposure level of AAB and GA of infants may cause different response to AAB exposure in these infants. Our findings merit further prospective studies with predesigned specific variables to assess the effect of AAB in a subgroup of infants with a narrower GA range.

## Data Availability Statement

The datasets analyzed in this study are available from the corresponding authors on a reasonable request. Requests to access these datasets should be directed to Liya Ma, maliya226@qq.com, or Ping-Ping Jiang, jiangpp3@mail.sysu.edu.cn.

## Author Contributions

P-PJ and LM: conceptualization, supervision, and project administration. P-PJ, PL, and KZ: methodology. PL: software, formal analysis, writing—original draft preparation, and visualization. KZ, YC, XG, LL, and PZ: data validation. PL and XG: investigation. KZ, YC, PZ, and LM: resources. TW, LL, and XG: data curation. P-PJ: writing—review and editing and funding acquisition. All authors have read and agreed to the published version of the manuscript.

## Funding

This work was financially supported by a starting grant (Grant No. 2017181) to P-PJ from Sun Yat-Sen University, China and the Science, Technology and Innovation Commission of Shenzhen Municipality, China (JCYJ201908809183601667) to LM.

## Conflict of Interest

The authors declare that the research was conducted in the absence of any commercial or financial relationships that could be construed as a potential conflict of interest.

## Publisher's Note

All claims expressed in this article are solely those of the authors and do not necessarily represent those of their affiliated organizations, or those of the publisher, the editors and the reviewers. Any product that may be evaluated in this article, or claim that may be made by its manufacturer, is not guaranteed or endorsed by the publisher.

## References

[1] de Jonge L Bos HJ van Langen IM de Jong-van den Berg LT Bakker MK. Antibiotics prescribed before, during and after pregnancy in the Netherlands: a drug utilization study. Pharmacoepidemiol Drug Saf. (2014) 23:60–8. 10.1002/pds.3492 23913654

[2] Zimmermann P Curtis N. Effect of intrapartum antibiotics on the intestinal microbiota of infants: a systematic review. Arch Dis Child Fetal Neonatal Ed. (2020) 105:201–8. 10.1136/archdischild-2018-316659 31296695

[3] Örtqvist AK Lundholm C Halfvarson J Ludvigsson JF Almqvist C. Fetal and early life antibiotics exposure and very early onset inflammatory bowel disease: a population-based study. Gut. (2019) 68:218–25. 10.1136/gutjnl-2017-314352 29321166

[4] Chu S Yu H Chen Y Chen Q Wang B Zhang J. Periconceptional and gestational exposure to antibiotics and childhood asthma. PLoS ONE. (2015) 10:e0140443. 10.1371/journal.pone.0140443 26488397 PMC4619063

[5] Mueller NT Whyatt R Hoepner L Oberfield S Dominguez-Bello MG Widen EM. Prenatal exposure to antibiotics, cesarean section and risk of childhood obesity. Int J Obes. (2015) 39:665–70. 10.1038/ijo.2014.180 25298276 PMC4390478

[6] Kenyon SL Taylor DJ Tarnow-Mordi W Group OC. Broad-spectrum antibiotics for preterm, prelabour rupture of fetal membranes: the ORACLE I randomised trial. ORACLE Collaborative Group Lancet. (2001) 357:979–88. 10.1016/S0140-6736(00)04233-1 11293640

[7] Weintraub AS Ferrara L Deluca L Moshier E Green RS Oakman E. Antenatal antibiotic exposure in preterm infants with necrotizing enterocolitis. J Perinatol. (2012) 32:705–9. 10.1038/jp.2011.180 22157626

[8] Nogacka AM Salazar N Arboleya S Suárez M Fernández N Solís G. Early microbiota, antibiotics and health. Cell Mol Life Sci. (2018) 75:83–91. 10.1007/s00018-017-2670-2 28988290 PMC11105232

[9] Chong CYL Bloomfield FH O'Sullivan JM. Factors affecting gastrointestinal microbiome development in neonates. Nutrients. (2018) 10:274. 10.3390/nu10030274 29495552 PMC5872692

[10] Ford SL Lohmann P Preidis GA Gordon PS O'Donnell A Hagan J. Improved feeding tolerance and growth are linked to increased gut microbial community diversity in very-low-birth-weight infants fed mother's own milk compared with donor breast milk. AmJ Clin Nutr. (2019) 109:1088–97. 10.1093/ajcn/nqz006 30982856 PMC6462428

[11] NEOVITA Study Group. Timing of initiation, patterns of breastfeeding, and infant survival: prospective analysis of pooled data from three randomised trials. Lancet Global Health. (2016) 4:e266–e75. 10.1016/S2214-109X(16)00040-1 27013313

[12] Karagol BS Zenciroglu A Okumus N Polin RA. Randomized controlled trial of slow vs rapid enteral feeding advancements on the clinical outcomes of preterm infants with birth weight 750–1250 g. J Parenteral Enteral Nutrition. (2013) 37:223–8. 10.1177/0148607112449482 22664861

[13] Krishnamurthy S Gupta P Debnath S Gomber S. Slow versus rapid enteral feeding advancement in preterm newborn infants 1000-1499 g: a randomized controlled trial. Acta Paediatr. (2010) 99:42–6. 10.1111/j.1651-2227.2009.01519.x 20002013

[14] Waard M Li Y Zhu Y Ayede AI Berrington J Bloomfield FH. Time to full enteral feeding for very low-birth-weight infants varies markedly among hospitals worldwide but may not be associated with incidence of necrotizing enterocolitis: The NEOMUNE-NeoNutriNet Cohort Study. J Parenteral Enteral Nutr. (2019) 43:658–67. 10.1002/jpen.1466 30465333 PMC6531355

[15] Villar J Giuliani F Barros F Roggero P Coronado Zarco IA Rego MAS. Monitoring the postnatal growth of preterm infants: a paradigm change. Pediatrics. (2018) 141:e20172467. 10.1542/peds.2017-2467 29301912

[16] Zea-Vera A Ochoa TJ. Challenges in the diagnosis and management of neonatal sepsis. J Trop Pediatr. (2015) 61:1–13. 10.1093/tropej/fmu079 25604489 PMC4375388

[17] Hutzal CE Boyle EM Kenyon SL Nash JV Winsor S Taylor DJ. Use of antibiotics for the treatment of preterm parturition and prevention of neonatal morbidity: a metaanalysis. Am J Obstet Gynecol. (2008) 199:620.e1–8. 10.1016/j.ajog.2008.07.008 18973872

[18] Kenyon S Boulvain M Neilson JP. Antibiotics for preterm rupture of membranes. Cochr Database Syst Rev. (2013) 12:CD001058. 10.1002/14651858.CD001058.pub3 24297389 PMC11297390

[19] Kuzma-O'Reilly B Duenas ML Greecher C Kimberlin L Mujsce D Miller D. Evaluation, development, and implementation of potentially better practices in neonatal intensive care nutrition. Pediatrics. (2003) 111:e461–70. 12671166

[20] Patel AL Engstrom JL Meier PP Jegier BJ Kimura RE. Calculating postnatal growth velocity in very low birth weight (VLBW) premature infants. J Perinatol. (2009) 29:618–22. 10.1038/jp.2009.55 19461590 PMC2767524

[21] Fenton TR Kim JH. A systematic review and meta-analysis to revise the Fenton growth chart for preterm infants. BMC Pediatr. (2013) 13:59. 10.1186/1471-2431-13-59 23601190 PMC3637477

[22] Hornik CP Benjamin DK Becker KC Benjamin DK Li J Clark RH. Use of the complete blood cell count in early-onset neonatal sepsis. Pediatric Infect Dis J. (2012) 31:799–802. 10.1097/INF.0b013e318256905c 22531231 PMC3399972

[23] Murphy K Weiner J. Use of leukocyte counts in evaluation of early-onset neonatal sepsis. Pediatric Infect Dis J. (2012) 31:16–9. 10.1097/INF.0b013e31822ffc17 21860335

[24] Guida JD Kunig AM Leef KH McKenzie SE Paul DA. Platelet count and sepsis in very low birth weight neonates: is there an organism-specific response? Pediatrics. (2003) 111:1411–5. 10.1542/peds.111.6.1411 12777561

[25] Hofer N Zacharias E Müller W Resch B. An update on the use of C-reactive protein in early-onset neonatal sepsis: current insights and new tasks. Neonatol. (2012) 102:25–36. 10.1159/000336629 22507868

[26] Wacker C Prkno A Brunkhorst FM Schlattmann P. Procalcitonin as a diagnostic marker for sepsis: a systematic review and meta-analysis. Lancet Infect Dis. (2013) 13:426–35. 10.1016/S1473-3099(12)70323-7 23375419

[27] Amorim LD Cai J. Modelling recurrent events: a tutorial for analysis in epidemiology. Int J Epidemiol. (2015) 44:324–33. 10.1093/ije/dyu222 25501468 PMC4339761

[28] Cantey JB Pyle AK Wozniak PS Hynan LS Sánchez PJ. Early antibiotic exposure and adverse outcomes in preterm, very low birth weight infants. J Pediatr. (2018) 203:62–7. 10.1016/j.jpeds.2018.07.036 30172430

[29] The R Core Team. R: A Language and Environment for Statistical Computing. Vienna, Austria: R Foundation for Statistical Computing. (2013).

[30] The R. StudioTeam. R Studio: Integrated Development for R 402. ed Boston, MA, USA: RStudio, Inc. (2019).

[31] Therneau TM Grambsch PM. Modeling Survival Data: Extending the Cox Model. 1 ed: Springer. (2000). 10.1007/978-1-4757-3294-8_1

[32] Abiramalatha T Thanigainathan S Ninan B. Routine monitoring of gastric residual for prevention of necrotising enterocolitis in preterm infants. Cochr Database Syst Rev. (2019) 7:CD012937. 10.1002/14651858.CD012937.pub2 31425604 PMC6699661

[33] de Greeff A Schokker D Roubos-van den Hil P Ramaekers P Vastenhouw SA Harders F. The effect of maternal antibiotic use in sows on intestinal development in offspring. J Animal Sci. (2020) 98:1–13. 10.1093/jas/skaa181 32479635 PMC7295330

[34] Gonzalez-Perez G Hicks AL Tekieli TM Radens CM Williams BL Lamousé-Smith ESN. Maternal antibiotic treatment impacts development of the neonatal intestinal microbiome and antiviral immunity. J Immun. (2016) 196:3768–79. 10.4049/jimmunol.1502322 27036912

[35] Reed BD Schibler KR Deshmukh H Ambalavanan N Morrow AL. The Impact of maternal antibiotics on neonatal disease. J Ped. (2018) 197:97–103.e3. 10.1016/j.jpeds.2018.01.056 29551319 PMC6028045

[36] Fanaro S. Feeding intolerance in the preterm infant. Early Hum Dev. (2013) 89:S13–20. 10.1016/j.earlhumdev.2013.07.013 23962482

[37] Nguyen DN Thymann T Goericke-Pesch SK Ren S Wei W Skovgaard K. Prenatal intra-amniotic endotoxin induces fetal gut and lung immune responses and postnatal systemic inflammation in preterm pigs. Am J Pathol. (2018) 188:2629–43. 10.1016/j.ajpath.2018.07.020 30314768

[38] Chan GJ Lee ACC Baqui AH Tan J Black RE. Risk of early-onset neonatal infection with maternal infection or colonization: a global systematic review and meta-analysis. PLoS Med. (2013) 10:e1001502. 10.1371/journal.pmed.1001502 23976885 PMC3747995

[39] Thinkhamrop J Hofmeyr GJ Adetoro O Lumbiganon P Ota E. Antibiotic prophylaxis during the second and third trimester to reduce adverse pregnancy outcomes and morbidity. Cochr Database Syst Rev. (2015) 6:CD002250. 10.1002/14651858.CD002250.pub2 26092137 PMC7154219

[40] Miller JE Wu C Pedersen LH de Klerk N Olsen J Burgner DP. Maternal antibiotic exposure during pregnancy and hospitalization with infection in offspring: a population-based cohort study. Int J Epidemiol. (2018) 47:561–71. 10.1093/ije/dyx272 29415232

[41] Pereira-da-Silva L Virella D Fusch C. Nutritional assessment in preterm infants: a practical approach in the NICU. Nutrients. (2019) 11:1999. 10.3390/nu11091999 31450875 PMC6770216

[42] Watanabe Y Itabashi K Taki M Miyazawa T Nakano Y Murase M. Body length and occipitofrontal circumference may be good indicators of neurodevelopment in very low birthweight infants - secondary publication. Acta Paediatr. (2018) 107:975–80. 10.1111/apa.14250 29385636

[43] Umbrello M Elia G Destrebecq AL Iapichino G. Tolerance of enteral feeding: from quantity to quality of gastric residual volume? Intensive Care Med. (2009) 35:1651–2. 10.1007/s00134-009-1525-1 19526217

[44] Dutta S Reddy R Sheikh S Kalra J Ray P Narang A. Intrapartum antibiotics and risk factors for early onset sepsis. Arch Dis Child Fetal Neonatal Ed. (2010) 95:F99–103. 10.1136/adc.2009.163220 19996327

[45] Tanaka S Tsumura K Nakura Y Tokuda T Nakahashi H Yamamoto T. New antibiotic regimen for preterm premature rupture of membrane reduces the incidence of bronchopulmonary dysplasia. J Obstet Gynaecol Res. (2019) 45:967–73. 10.1111/jog.13903 30687995

