# Supplementary material for: Antenatal Antibiotic Exposure Affects Enteral Feeding, Body Growth, and Neonatal Infection in Preterm Infants: A Retrospective Study
Source: Front Pediatr. 2021 Dec 22;9:750058. doi: 10.3389/fped.2021.750058 (PMC8727690; doi:10.3389/fped.2021.750058)
Supplement: Supplementary file 1 [file Data_Sheet_1.docx]

Supplementary Material

**Table S1.** Treatment Protocol of Antenatal Antibiotics

| **Maternalindication** | **Antenatal antibiotics** |
| --- | --- |
| Cesarean prophylaxis | Cefazolin 2g half an hour before surgery and 1g at 6 and 12 hours after surgery |
| GBS prophylaxis | 480 IU of penicillin was given as the first dose when the membranes were broken or regular uterine contractions were observed, followed by 240 IU of penicillin intravenously every 4 hours until delivery. If the pregnant woman was allergic to penicillin, sensitive antibiotic was administrated according to the results of drug sensitivity test |
| PPROM | Cefazolin 2g intravenously per 8 hours until delivery when prolonged rupture of membranes over 12 hours |

GBS, group B streptococcus; PPROM, preterm premature rupture of membranes.

**Table S2.** Diagnosing Criteria of Adverse Neonatal Outcomes and Adverse Maternal Conditions

| **Outcomes / Conditions** | **Diagnosis** | **References** |
| --- | --- | --- |
| NEC | stage II or greater according to Bell’s criteria | Bell, M.J., et al., Ann Surg, 1978. 187(1): p. 1-7. |
| RDS | occurrence of respiratory distress within the first 24 h of life and presence of typical chest X-ray findings | De Luca, D., et al., Lancet Respir Med, 2017. 5(8): p. 657-666. |
| BPD | requirement for oxygen at postmenstrual age of 36 weeks or at the time of transfer to a level II facility | Dd |
| ROP | defined according to the International Classification of ROP | The International Classification of Retinopathy of Prematurity revisited. Arch Ophthalmol, 2005. 123(7): p. 991-9. |
| Sepsis | culture-proven sepsis and clinical sepsis without culture evidence | Bedford Russell AR. Paediatrics Child Health, 2011. 21(6): 265-269. |
| Brain injury | grade 3 or 4 intraventricular hemorrhage according to the criteria of Papile et al or periventricular leukomalacia, diagnosed by cranial ultrasonography. | Papile, L.A., et al., J Pediatr, 1978. 92(4): p. 529-34. |
| Chorioamnionitis | an infection with resultant inflammation of any combination of the amniotic fluid, placenta, fetus, fetal membranes, or decidua, including isolated maternal fever, suspected intraamniotic infection and confirmed intraamniotic infection | Committee Opinion No. 712. Obstet Gynecol, 2017. 130(2): e95-e101 |
| PPROM | rupture of membranes before labor that occurs before 37 weeks of gestation | ACOG Practice Bulletin No. 188: Obstet Gynecol, 2018. 131(1): p. e1-e14. |
| Maternal diabetes | diagnosed in the second or thirdtrimester of pregnancy that was not clearly overt diabetes prior to gestation and pregestational diabetes mellitus diabetes | American Diabetes, A., 2. Diabetes Care, 2019. 42(Suppl 1): p. S13-S28. |
| Maternal hypertension | including gestational hypertension, preeclampsia, eclampsia, chronic hypertension with superimposed preeclampsia and chronic hypertension | Hypertension in pregnancy. Obstet Gynecol, 2013. 122(5): p. 1122-31. |

BPD, bronchopulmonary dysplasia; NEC, necrotizing enterocolitis; PPROM, preterm premature rupture of membranes; RDS, respiratory distress syndrome; ROP, retinopathy of prematurity.

**Table S3.** Maternal Characteristics of the Study Population

| **Characteristics** | **No AAB Group**  **(n = 1,505)** | **AAB Group**  **(n = 1,038)** | **P** |
| --- | --- | --- | --- |
| Age, y, mean ± SD | 30.7 ± 4.8 | 30.7 ± 4.8 | 0.89^a^ |
| Tertiary education ^c^, yes, n (%)  (n = 1,505 vs n = 1,037) | 876 (58.2) | 601 (58.0) | 0.93^b^ |
| Nulliparity, yes, n (%) | 768 (51.0) | 544 (52.4) | 0.52^b^ |
| Assisted reproduction, yes, n (%) | 169 (11.2) | 168 (16.2) | **<0.001^b^** |
| Cesarean delivery, yes, n (%) | 937 (62.3) | 653 (62.9) | 0.77^b^ |
| ACS,yes,n (%) | 982 (65.2) | 915 (88.2) | **<0.001^b^** |
| Maternal infection, yes, n (%) | 569 (37.8) | 642 (61.8) | **<0.001^b^** |
| Chorioamnionitis, yes, n (%) | 289 (19.2) | 389 (37.5) | **<0.001^b^** |
| PPROM, yes, n (%) | 393 (26.1) | 555 (53.5) | **<0.001**^b^ |
| Maternal diabetes, yes, n (%) | 353 (23.5) | 242 (23.3) | 0.97^b^ |
| Maternal hypertension, yes, n (%) | 312 (20.7) | 122 (11.8) | **<0.001^b^** |

^a^ tested with Student’s *t* test. ^b^ tested with Chi-square test. ^c^ data missing in tertiary education. AAB, antenatal antibiotics; ACS, antenatal corticosteroids; PPROM, preterm premature rupture of membranes; SD, standard deviation.

**Table S4.** Neonatal OutcomesRelated to Enteral Feeding, Body Growth, and Infection by DOTs of AAB Exposure

| **Outcomes** | **≤3 d**  **(n = 741)** | **>3 d**  **(n = 297)** | **P** |
| --- | --- | --- | --- |
| Feeding intolerance, yes, n (%) | 130 (17.5) | 131 (44.1) | **<0.001^c^** |
| **Enteral feeding** | | | |
| TIEF, d, median (IQR) | 1.0 (1.0–2.0) | 3.0 (1.0–6.0) | **<0.001^a^** |
| Advancement rate of enteral feeding ^d^, mL/kg/d, mean ± SD,  (n = 735 vs n = 295) | 16.6 ± 6.2 | 13.1 ± 6.3 | **<0.001^b^** |
| Attaining full enteral feeding before discharge, yes, n (%) | 475 (64.1) | 253 (85.2) | **<0.001^c^** |
| TFEF120, d, median (IQR) | 6.0 (5.0–8.0) | 7.0 (6.0–13.0) | **<0.001^a^** |
| **Body growth** | | | |
| Regaining birth weight before discharge, yes, n (%) | 428 (57.8) | 250 (84.2) | **<0.001^c^** |
| TRBW, d, median (IQR) | 7.0 (4.0–9.0) | 7.0 (3.0–11.0) | **<0.05^a^** |
| Weight velocity, g/kg/d, mean ± SD | -0.2 ± 7.3 | 2.9 ± 6.0 | **<0.001^b^** |
| Bodyweight Z-score at birth,  mean ± SD | -0.4 ± 0.8 | 0.0 ± 0.6 | **<0.001^b^** |
| Bodyweight Z-score on discharge, mean ± SD | -1.3 ± 0.8 | -1.2 ± 0.7 | 0.06^b^ |
| Δbodyweight Z-score, mean ± SD | -0.8 ± 0.5 | -1.2 ± 0.6 | **<0.001^b^** |
| **Infection** | | | |
| Sepsis, yes, n (%) | 40 (5.4) | 50 (16.8) | **<0.001^c^** |
| Clinical infection, yes, n (%) | 33 (4.5) | 39 (13.1) | **0.001^c^** |
| Time to 1st infection, d,  median (IQR), (n = 33 vs n = 39) | 4.0 (2.0–11.0) | 5.0 (1.0–16.0) | 0.67^a^ |
| Clinical infection episodes, times, median (IQR), (n = 33 vs n = 39) | 1.0 (1.0–2.0) | 1.0 (1.0–2.0) | 0.62^a^ |

^a^ tested with Wilcoxon rank sum test. ^b^ tested with Student’s *t* test.^c^ tested with Chi-square test.^d^ 8 subjects discharged when TIEF was initiated and the rate of enteral feeding advancement could not be calculated. AAB, antenatal antibiotics; DOT, days of treatment;IQR, interquartile range;SD, standard deviation;TFEF120, time to full enteral feeding of 120 mL/kg/d; TIEF, time to the initiation of enteral feeding; TRBW, time to regain birth weight.

**Table S5**. Neonatal OutcomesRelated to Enteral Feeding, Body Growth, and Infection of No AAB and AAB Group - Stratified Analysis by GA

| **Outcomes** | **<34 weeks (n = 929)** | | **p** | **≥34 weeks (n = 1,614)** | | **p** |
| --- | --- | --- | --- | --- | --- | --- |
|  | **No AAB Group**  **(n = 467)** | **AAB Group**  **(n = 462)** |  | **No AAB Group**  **(n = 1,038)** | **AAB Group**  **(n = 576)** |  |
| Feeding intolerance,  yes, n (%) | 255 (54.6) | 235 (50.9) | 0.28^c^ | 74 (7.1) | 26 (4.5) | **<0.05^c^** |
| **Enteral feeding** | | | | | | |
| TIEF, d, median (IQR) | 4.0 (2.0–7.5) | 3.0 (2.0–7.0) | 0.44^a^ | 1.0 (1.0–2.0) | 1.0 (1.0–1.0) | **0.001^a^** |
| Advancement rate of enteral feeding ^d^, mL/kg/d,  mean ± SD | 12.6 ± 5.9 | 12.2 ± 6.1 | 0.36^b^ | 18.4 ± 5.9 | 18.4 ± 5.3 | 0.66^b^ |
| Attainment of full enteral feeding before discharge,  yes, n (%) | 440 (94.2) | 426 (92.2) | 0.28^c^ | 557 (53.7) | 302 (52.4) | 0.67^c^ |
| TFEF120, d, median (IQR) | 8.0 (6.0–13.0) | 9.0 (6.0–13.0) | 0.31^a^ | 5.0 (4.0–7.0) | 5.0 (4.0–6.0) | 0.54^a^ |
| **Body growth** | | | | | | |
| Regaining birth weight before discharge, yes, n (%) | 409 (87.6) | 433 (93.7) | **<0.01^c^** | 531 (51.2) | 245 (42.5) | **0.001^c^** |
| TRBW, d, median (IQR) | 9.0 (5.0–12.0) | 8.0 (4.0–11.0) | **<0.001**^a^ | 6.0 (4.0–8.0) | 6.0 (4.0–8.0) | **0.04^a^** |
| Weight velocity, g/kg/d,  mean ± SD | 2.8 ± 6.7 | 4.1 ± 5.4 | **0.001^b^** | -1.8 ± 6.0 | -2.1 ± 7.1 | 0.54^b^ |
| Bodyweight Z-score at birth,  mean ± SD | -0.2 ± 0.8 | 0.0 ± 0.7 | **<0.01^b^** | -0.5 ± 0.8 | -0.5 ± 0.8 | 0.41^b^ |
| Bodyweight Z-score on discharge, mean ± SD | -1.5 ± 0.9 | -1.3 ± 0.8 | **<0.001**^b^ | -1.2 ± 0.9 | -1.3 ± 0.8 | 0.84^b^ |
| Δbodyweight Z-score,  mean ± SD | -1.3 ± 0.6 | -1.2 ± 0.6 | 0.05^b^ | -0.7 ± 0.3 | -0.7 ± 0.4 | 0.16^b^ |
| **Infection** | | | | | | |
| Sepsis, yes, n (%) | 71 (15.2) | 82 (17.7) | 0.34^c^ | 27 (2.6) | 8 (1.4) | 0.15^c^ |
| Clinical infection, yes, n (%) | 67 (14.3) | 64 (13.9) | 0.90^c^ | 28 (2.7) | 8 (1.4) | 0.13^c^ |
| Time to 1st infection, d, median (IQR) | 3.0 (1.0–7.5) | 4.0 (2.0–13.5) | 0.09^a^ | 3.0 (1.8–5.0) | 3.5 (3.0–4.5) | 0.56^a^ |
| Clinical infection episodes, times, median (IQR) | 1.0 (1.0–2.0) | 1.0 (1.0–2.0) | 0.20^a^ | 1.0 (1.0–1.0) | 1.0 (1.0–1.0) | 0.92^a^ |

^a^ tested with Wilcoxon rank sum test. ^b^ tested with Student’s *t* test.^c^ tested with Chi-square test.^d^23 subjects discharged when TIEF was initiated and the rate of enteral feeding advancement could not be calculated. AAB, antenatal antibiotics; GA, gestational age;IQR, interquartile range;SD, standard deviation; TFEF120, time to full enteral feeding of 120 mL/kg/d; TIEF, time to the initiation of enteral feeding; TRBW, time to regain birth weight.
